# Spatial pattern of isoniazid-resistant tuberculosis and its associated factors among a population with migrants in China: a retrospective population-based study

**DOI:** 10.3389/fpubh.2024.1372146

**Published:** 2024-03-06

**Authors:** Hongyin Zhang, Ruoyao Sun, Zheyuan Wu, Yueting Liu, Meiru Chen, Jinrong Huang, Yixiao Lv, Fei Zhao, Yangyi Zhang, Minjuan Li, Hongbing Jiang, Yiqiang Zhan, Jimin Xu, Yanzi Xu, Jianhui Yuan, Yang Zhao, Xin Shen, Chongguang Yang

**Affiliations:** ^1^School of Public Health (Shenzhen), Shenzhen Campus of Sun Yat-sen University, Shenzhen, Guangdong, China; ^2^Division of TB and HIV/AIDS Prevention, Shanghai Municipal Center for Disease Control and Prevention, Shanghai, China; ^3^Shanghai Institutes of Preventive Medicine, Shanghai, China; ^4^Department of Pharmacy, Beijing Hospital, National Center of Gerontology, Beijing, China; ^5^Institute of Geriatric Medicine, Chinese Academy of Medical Sciences, Beijing, China; ^6^Beijing Key Laboratory of Assessment of Clinical Drugs Risk and Individual Application (Beijing Hospital), Beijing, China; ^7^Department of Epidemiology, School of Public Health and Key Laboratory of Public Health Safety, Fudan University, Shanghai, China; ^8^Nanshan District Center for Disease Control and Prevention, Shenzhen, Guangdong, China; ^9^Department of Epidemiology of Microbial Diseases, Yale School of Public Health, New Haven, CT, United States

**Keywords:** isoniazid resistance, spatial analysis, migrant, China, tuberculosis

## Abstract

**Background:**

Isoniazid-resistant, rifampicin-susceptible tuberculosis (Hr-TB) globally exhibits a high prevalence and serves as a potential precursor to multidrug-resistant tuberculosis (MDR-TB). Recognizing the spatial distribution of Hr-TB and identifying associated factors can provide strategic entry points for interventions aimed at early detection of Hr-TB and prevention of its progression to MDR-TB. This study aims to analyze spatial patterns and identify socioeconomic, demographic, and healthcare factors associated with Hr-TB in Shanghai at the county level.

**Method:**

We conducted a retrospective study utilizing data from TB patients with available Drug Susceptible Test (DST) results in Shanghai from 2010 to 2016. Spatial autocorrelation was explored using Global Moran’s I and Getis-Ord $Gi^{*}$ statistics. A Bayesian hierarchical model with spatial effects was developed using the INLA package in R software to identify potential factors associated with Hr-TB at the county level.

**Results:**

A total of 8,865 TB patients with DST were included in this analysis. Among 758 Hr-TB patients, 622 (82.06%) were new cases without any previous treatment history. The drug-resistant rate of Hr-TB among new TB cases in Shanghai stood at 7.20% (622/8014), while for previously treated cases, the rate was 15.98% (136/851). Hotspot areas of Hr-TB were predominantly situated in southwestern Shanghai. Factors positively associated with Hr-TB included the percentage of older adult individuals (RR = 3.93, 95% Crl:1.93–8.03), the percentage of internal migrants (RR = 1.35, 95% Crl:1.15–1.35), and the number of healthcare institutions per 100 population (RR = 1.17, 95% Crl:1.02–1.34).

**Conclusion:**

We observed a spatial heterogeneity of Hr-TB in Shanghai, with hotspots in the Songjiang and Minhang districts. Based on the results of the models, the internal migrant population and older adult individuals in Shanghai may be contributing factors to the emergence of areas with high Hr-TB notification rates. Given these insights, we advocate for targeted interventions, especially in identified high-risk hotspots and high-risk areas.

## 1 Introduction

Tuberculosis (TB), an infectious disease caused by *Mycobacterium tuberculosis*, remains a significant global public health concern. In 2022, it was reported that approximately 10.6 million individuals fell ill with tuberculosis, with China ranking as the third highest burden country worldwide (1). The emergence of drug-resistant TB poses substantial challenges to TB control efforts, because of its prolonged treatment regimens, less favorable outcomes, and increased financial burden (2, 3). Notably, isoniazid-resistant TB, without concurrent rifampicin resistance (Hr-TB) should be paid attention to. It is estimated to occur in 7.4% of new TB patients and 11.4% of previously treated TB patients globally (4). Moreover, studies have shown that there is a likelihood of its progression to multidrug-resistant TB (MDR-TB) (5–8). However, Isoniazid-resistant TB including Hr-TB, is receiving less attention compared with other forms of drug-resistant TB (4, 9, 10). Ignoring Isoniazid-resistant TB may lead these patients to have worse outcomes and promote them to become MDR-TB.

A substantial number of isoniazid-resistant TB is undiagnosed in the population (11). A more effective strategy should be implemented to control this form of TB. Analyzing hotspot areas of a disease and identifying the potential factors associated with the disease could enhance our understanding of it, and further help medical institutes to take a better control strategy (12). Previous research revealed that isoniazid-resistant tuberculosis patients were clustered in specific geographical areas (13). Currently, the understanding of the potential factors associated with isoniazid-resistant TB is limited. Meanwhile, previous research has highlighted the emergence of TB is associated with socio-economic, demographic, and other factors in China employing Bayesian hierarchical models (14–16).

Shanghai, as a metropolis with a substantial internal migrant population in China, faces the intricate challenge of managing TB and simultaneously facing medical pressure from the neighboring smaller cities. Given this situation, implementing effective TB control measures is crucial in Shanghai. Despite efforts, the incidence rate of TB in Shanghai remains above 22 cases per 100,000 population as of 2021 (17). Currently, our understanding of the notification rates, spatial patterns, and influenced factors of Hr-TB is limited. To provide a strategic entry point for managing Hr-TB, a retrospective study was conducted in Shanghai, China. This study aims to analyze the demographic characteristics and the spatial pattern of isoniazid-resistant TB and identify the underlying factors of isoniazid-resistant TB.

## 2 Materials and methods

### 2.1 Study settings

Shanghai is a metropolis with a population of 24 million and a total area of 6,340 km^2^ in 2016. It encompasses 16 districts and 214 counties. The study excluded Chongming Island due to its distant location from the central city area.

### 2.2 Data source and definitions

The datasets for this study were acquired from a routine surveillance system operated by the Shanghai Municipal Center for Disease Control and Prevention. These datasets included routine demographic information of tuberculosis (TB) patients and the Drug Susceptibility Tests (DST) outcomes for first-line drugs. The data spanned the period from 2010 to 2016. Socio-economic, demographic and healthcare factors in Shanghai were collected from a city-wide census and the Shanghai statistical yearbook (18, 19). In this research, the diagnosis delay time was defined as the period between the emergence of the first symptom(s) possibly linked to TB and the date when the patient received their initial pulmonary TB diagnosis. The sources of TB patients were classified into two distinct groups: active screening, which encompassed contact tracing investigations and health examinations; and passive screening, which included symptom-based visits and referrals by community healthcare centers or non-TB-designated facilities.

### 2.3 Statistical analysis

#### 2.3.1 Demographic characters

The focus of this study was on two forms of TB: isoniazid-resistant, rifampicin-susceptible TB (Hr-TB) and drug-susceptible TB (DS-TB). Proportions between the Hr-TB and DS-TB were compared using the Chi-squared test or *Fisher* exact test. Statistical significance was established at a level of 0.01 or less. To identify the risk factors associated with Hr-TB, both univariable and multivariable logistic regression analyses were employed. Variables with a *p*-value < 0.01 were considered independent risk factors. The statistical analysis was performed using R (version 4.2.1).

#### 2.3.2 Spatial analysis

The addresses of TB patients were aggregated at the county level, and the notification rates of Hr-TB were calculated. Spatial autocorrelation analysis was then conducted to investigate the spatial distribution of TB notification rates. Spatial autocorrelation can be divided into global and local spatial autocorrelation (20). In this study, we employed a Global Moran’s I analysis to assess global autocorrelation and explore the overall distribution characteristics of the study area. (21, 22). Then Getis-Ord $Gi^{*}$ index was employed to assess local autocorrelation and identify cold spots or hot spots of Hr-TB. (23, 24). These analyses were conducted using ArcGIS 10.7 software.

### 2.4 Spatial model

Spatial models incorporating socioeconomic, demographic, and healthcare covariates were constructed to analyze potential factors associated with Hr-TB. The potential associated with TB variables consisted of population density, the percentage of older adult people, the percentage of internal migrant people, the percentage of female people, the percentage of male people, the number of healthcare institutions per 100 population, the number of health technicians per 100 population, the number of hospital beds per 100 population, Household size, Gross Domestic Product *per capita*. Stepwise model selection was conducted to initially screen the variables and mitigate potential collinearity among the covariates within the model. To balance the simplicity and accuracy of model fitting, Akaike information criterion (AIC) was set as the selection criteria for inclusion or exclusion of variables (25). Collinearity was assessed through tolerance difference.

Firstly, we conducted a Geographically weighted regression (GWR). The equation for the GWR model is as follows (26):


$$y_{i}=\beta_{0}\left(\mu_{i},\nu_{i}\right)+\sum_{k=1}^{p}\beta_{k}\left(\mu_{i},\nu_{i}\right)x_{ik}+\varepsilon_{i}\left(i=1,2,\ldots n\right)$$

Where $y_{i}$ is the number of TB cases at the *i*th sample point. $\left(\mu_{i},\nu_{i}\right)$ is the spatial coordinate of sample point i. $\beta_{0}\left(\mu_{i},\nu_{i}\right)$ represents the regression constant, while $\beta_{k}\left(\mu_{i},\nu_{i}\right)$ denotes the regression coefficient for sample point i. $\varepsilon_{i}$ is the random error. $x_{ik}$ is the kth potential factors for the ith sample point. The GWR model was built with spgwr package in R software version 4.2.3.

Furthermore, four Bayesian hierarchical models were implemented using the INLA package (www.r-inla.org) in R software version 4.2.3 (27), following the approach proposed by Paula Moraga (28). It was assumed that the observed counts of TB were independently distributed according to a Poisson distribution in the model and the model is as follows:


$$Y_{i}\sim \text{Poisson}\left(E_{i}\theta_{i}\right)\;,$$

where $Y_{i}$ denotes the number of notified TB cases, $E_{i}$ is the expected count and $\theta_{i}$ is the relative risk in area i. The mathematical forms of the four models are as follows:

Non-spatial effect model: $\log\left(\theta_{i}\right)=\beta_{0}+\sum_{j=1}^{k}\beta_{j}X_{ji},$

Spatial structured effect model: $\log\left(\theta_{i}\right)=\beta_{0}+\sum_{j=1}^{k}\beta_{j}X_{ji}+u_{i},$

Spatial unstructured effect model: $\;\log\left(\theta_{i}\right)=\beta_{0}+\sum_{j=1}^{k}\beta_{j}X_{ji}+v_{i},$

Spatial structured and unstructured effect model: $\log\left(\theta_{i}\right)=\beta_{0}+\sum_{j=1}^{k}\beta_{j}X_{ji}+u_{i}+v_{i}$,

where $\beta_{0}$ represents the intercept of the overall risk, $\beta_{j}$ denotes the coefficient of the covariates for i=1,…,n;j=1,…,k. We specify $u_{i}$ is a spatial structured component modeled with a CAR distribution, $u_{i}|u_{-i}\sim N\left({\bar{u}}_{\delta_{i}},\frac{\sigma_{u}^{2}}{n\delta_{i}}\right)$, and $v_{i}$ is an unstructured spatial effect defined as $v_{i}\sim N\left(0,\sigma_{v}^{2}\right)$. The relative risk (RR), denoted as $\theta_{i}$, quantifies whether an area has a higher ($\theta_{i}$> 1) or lower ($\theta_{i}$< 1) risk compared to the average risk in the standard population. To establish a suitable model, four models with different effects were built and were selected with the deviance information criterion (DIC). Conditional Predictive Ordinate (CPO) was used to evaluate the model (29). Finally, we conducted a sensitivity analysis by changing the prior information of spatial effects in the final model.

## 3 Results

### 3.1 Demographic characteristics of Hr-TB

A total of 8,865 tuberculosis cases with available DST results were included in the analysis (Supplementary Figure S1). For the new TB cases in Shanghai, the drug-resistant rate of Hr-TB stood at 7.20% (622/8014), for previously treated TB cases, this rate was 15.98% (136/851). Among the 758 cases of Hr-TB, the majority were male (74.8%, 567/758) and the age group with the highest proportion of cases was 25–44 years (34.83%, 264/758; Table 1). Migrant cases accounted for over half of the Hr-TB cases (53.03%,402/758). The three main occupational groups among Hr-TB cases were labor workers (29.34%, 233/758), commercial services (23.19%, 163/758), and farmers (16.02%, 132/758; Table 1). Both new and previously treated Hr-TB cases were mainly detected in passive case findings (94.86 and 98.53%, respectively; Table 2). In addition, Hr-TB patients had more poor outcomes than DS-TB (12.93% vs. 10.02%). Both Hr-TB and DS-TB patients had a high proportion of diagnosis delay (73.22% vs. 73.83% with more than 2 weeks of delay, respectively).

**Table 1:** Demographic characteristics of Hr-TB and DS-TB in Shanghai.

	Hr-TB (758)		DS-TB (8107)		
	*n*	(%)	*n*	(%)	*p*-value
*Gender*					
Female	191	(25.20)	2,350	(28.99)	0.03
Male	567	(74.80)	5,757	(71.01)	
*Age*					
15–24	139	(18.34)	1850	(22.82)	<0.01
25–44	264	(34.83)	2,803	(34.58)	
45–65	239	(31.53)	2064	(25.46)	
> 65	116	(15.30)	1,390	(17.15)	
*Ethnic group*					
Non-Han Chinese	12	(1.58)	141	(1.74)	0.76
Han Chinese	745	(98.28)	7,966	(98.26)	
*Demographic attributes*					
Resident	356	(46.97)	3,605	(44.47)	0.19
Migrant	402	(53.03)	4,502	(55.53)	
*Occupations*					
Household/Unemployed	40	(4.38)	677	(8.35)	0.04
Cadres and staff	33	(5.23)	376	(4.64)	
Labor worker	233	(29.34)	2,391	(29.49)	
Farmer	132	(16.02)	1,301	(16.05)	
Commercial service	163	(23.19)	1,660	(20.48)	
Student and teacher	17	(2.95)	289	(3.56)	
Others	108	(14.67)	1,143	(14.10)	
Unknown	32	(4.22)	270	(3.33)	
*Treatment history*					
New	622	(82.06)	7,392	(91.18)	<0.01
Previously treated	136	(17.94)	715	(8.82)	
*Patient source*					
Active screening	33	(4.35)	387	(4.77)	0.61
Passive case-finding	724	(95.51)	7,714	(95.15)	
*Diagnosis delay*					
0-2 weeks (w)	203	(26.78)	2,122	(26.17)	0.52
2w-1 months (m)	217	(28.63)	2,215	(27.32)	
1-3 m	265	(34.96)	2,968	(36.61)	
3-6 m	53	(6.99)	513	(6.33)	
6 m-1 year	20	(2.64)	289	(3.56)	
Outcomes					
*Favorable outcomes*					
Cure	494	(65.17)	5,536	(68.29)	<0.01
Treatment completion	166	(21.90)	1759	(21.70)	
*Poor outcomes*					
Failure	17	(2.24)	60	(0.74)	
non-TB death	10	(1.32)	198	(2.44)	
TB death	9	(1.19)	71	(0.88)	
Loss to follow up	4	(0.53)	66	(0.81)	
Others	45	(5.94)	314	(3.87)	
Unknown	13	(1.72)	103	(1.27)	

Hr-TB, isoniazid-resistant, rifampicin-susceptible tuberculosis; DS-TB, drug-susceptible tuberculosis.

**Table 2:** Demographic characteristics of Hr-TB among new and previously treated TB cases in Shanghai.

	Hr-TB				
	New cases (622)		Retreated cases (136)		
	*n*	(%)	*n*	(%)	*p*-value
*Gender*					
Female	168	(27.01)	23	(16.91)	0.0140
Male	454	(72.99)	113	(83.09)	
*Age*					
15–24	131	(21.06)	8	(5.88)	<0.01
25–44	224	(36.01)	40	(29.41)	
45–65	187	(30.07)	52	(38.24)	
> 65	80	(12.86)	36	(26.47)	
*Ethnic group*					
non-Han Chinese	9	(1.45)	3	(2.21)	0.4595
Han Chinese	613	(98.55)	133	(97.79)	
*Demographic attributes*					
Resident	279	(44.86)	59	(43.38)	0.7542
Migrant	343	(55.14)	77	(56.62)	
*Occupations*					
Household/Unemployed	134	(21.54)	29	(21.32)	0.0325
Cadres and staff	31	(4.98)	2	(1.47)	
Labor worker	199	(31.99)	34	(25.00)	
Farmer	32	(5.15)	8	(5.88)	
Catering service	11	(1.78)	2	(1.47)	
Student and teacher	16	(2.57)	1	(0.74)	
Others	170	(27.33)	57	(41.91)	
Unknown	29	(4.66)	3	(2.21)	
*Patient source*					
Active screening	32	(5.14)	1	(0.74)	0.0414
Passive case-finding	590	(94.86)	134	(98.53)	
*Diagnosis delay*					
0–2 weeks (w)	164	(26.37)	39	(28.68)	0.1045
2w–1 months (m)	182	(29.26)	35	(25.74)	
1–3 m	221	(35.53)	44	(32.35)	
3–6 m	43	(6.91)	10	(7.35)	
6 m–1 year	12	(1.93)	8	(5.88)	

Hr-TB, isoniazid-resistant, rifampicin-susceptible tuberculosis.

Among the cases of Hr-TB, a higher proportion of patients (82.06%, 622/758) were new cases compare with patients with previous treatment history (17.94%, 136/758; Table 1). Among the 622 cases of new Hr-TB, 55.14% (343/622) were migrants (Supplementary Figure S2). The Hr-TB drug-resistant rate of migrant previously treated cases was highest in all age groups (Supplementary Figure S3). Univariate analysis showed that male gender, migrant status, the age groups of 25 to 44 and 45 to 65, occupation as a farmer, student, and teacher, a history of previous TB treatment, and a total diagnosis delay ranging from 6 months to 1 year were significantly associated with Hr-TB. Multivariable logistic regression analysis showed that a history of previous TB treatment (aOR, 2.18; 95%CI, 1.76–2.70) and age between 45 to 65 (aOR, 1.39; 95%CI, 1.09–1.76) were the risk factor for Hr-TB. By contrast, occupation as a farmer (aOR, 0.59; 95%CI, 0.41–0.86) was a protective factor for Hr-TB (Table 3).

**Table 3:** Univariable and multivariable logistic regression analysis of Hr-TB in Shanghai.

Characteristics	Univariable			Multivariable		
	OR	CI	*p*	aOR	CI	*p*
*Gender*						
Female						
Male	1.22	1.02–1.44	0.03			
*Demographic attributes*						
Resident						
Migrant	0.9	0.78–1.05	0.19			
*Age*						
15–24						
25–44	1.25	1.01–1.55	0.04	1.17	0.94–1.47	0.16
45–65	1.54	1.24–1.92	<0.01	1.39	1.09–1.76	<0.01
> 65	1.11	0.86–1.43	0.42	1.04	0.77–1.41	0.79
*Ethnic group*						
Non-Han Chinese						
Han Chinese	1.1	0.61–2.00	0.74			
*Occupations*						
Household/Unemployed						
Cadres and staff	0.89	0.6–1.32	0.57	0.99	0.67–1.47	0.96
Labor worker	0.99	0.8–1.22	0.94	1.05	0.85–1.31	0.63
Farmer	0.6	0.42–0.86	<0.01	0.59	0.41–0.86	<0.01
Student and teacher	0.56	0.34–0.94	0.03	0.80	0.47–1.36	0.40
Others	0.99	0.8–1.22	0.90	0.99	0.79–1.24	0.93
Unclear	1.21	0.81–1.8	0.36	1.21	0.81–1.82	0.35
*Treatment category*						
New						
Previously treated	2.27	1.85–2.77	<0.01	2.18	1.76–2.70	<0.01
*Diagnosis delay*						
0–2 weeks						
2 weeks–1 month	1.02	0.84–1.25	0.82			
1–3 month	0.93	0.77–1.13	0.48			
3–6 month	1.08	0.79–1.49	0.62			
6 month–1 year	0.72	0.45–1.16	0.18			

OR, odds ratio; aOR, ajusted odds ratio; CI, confidence interval.

### 3.2 Spatial analysis of Hr-TB in Shanghai

**Figure 1:**
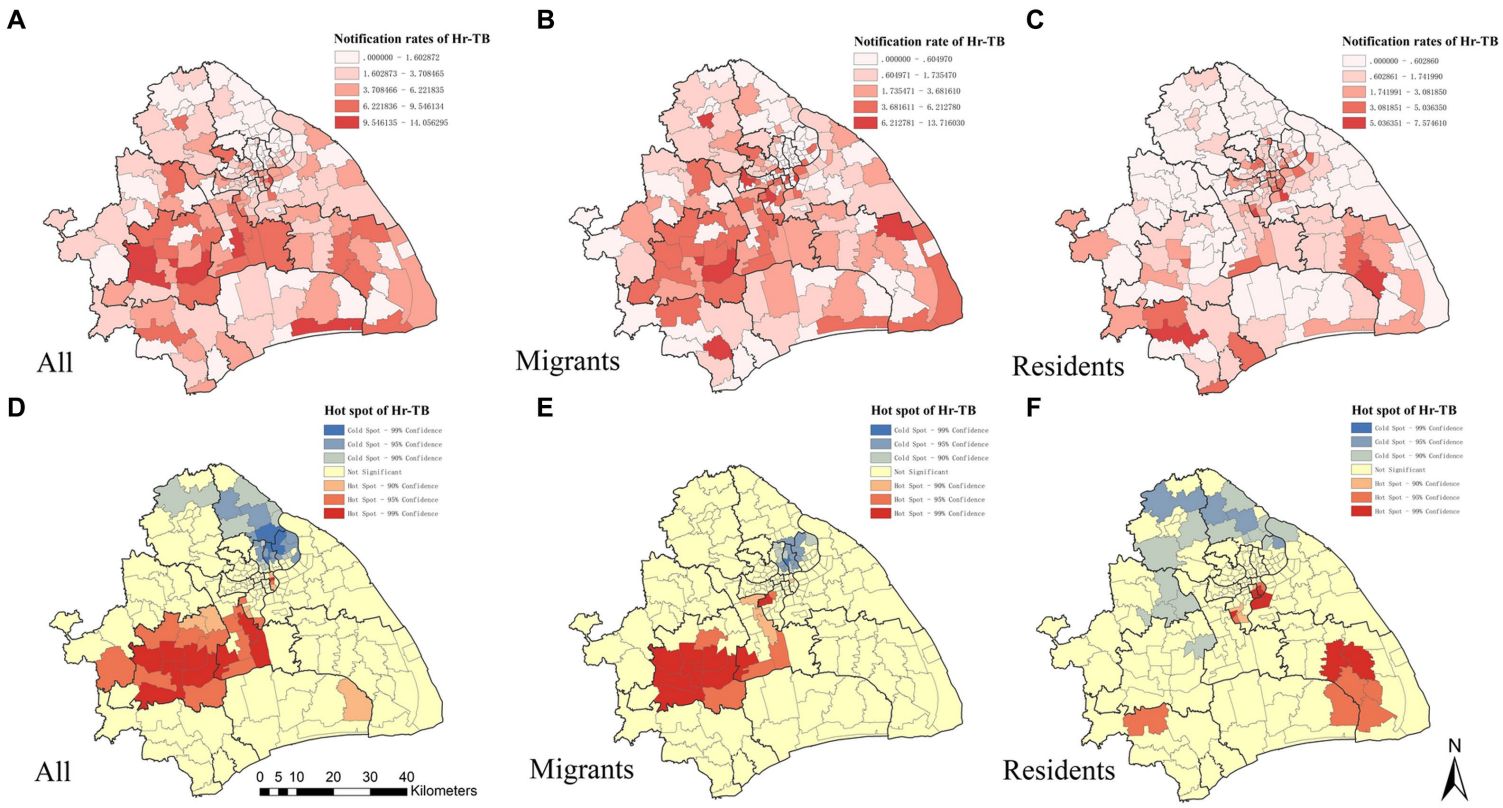
The spatial distribution and the hotspots analysis of Hr-TB by county level in Shanghai, 2010–2016. The geographic distribution of the notification rate of Hr-TB **(A)**, internal migrant Hr-TB **(B)**, resident Hr-TB **(C)**. Hotspot areas of Hr-TB **(D)**, internal migrant Hr-TB **(E)**, resident Hr-TB **(F)**. Hr-TB: Isoniazid-resistant, rifampicin-susceptible tuberculosis.

Overall, the notification rate for Hr-TB was at 3.43 per 100,000 population. We observed spatial variation in the Hr-TB notification rate across the city. The map in Figure 1A shows the spatial distribution of the Hr-TB notification rate in Shanghai at the county level. Seven of 196 counties have notification rates for Hr-TB greater than 10 per 100,000 population, five of those from Songjiang district and Minhang district. The global Moran’s I index for the Hr-TB notification rate was 0.22, and the Z score was 5.36 (*p* < 0.01), indicating the presence of significant, positive spatial autocorrelation in Hr-TB notification rate over the whole study area. A Getis-Ord $Gi^{*}$ analysis was conducted to further pinpoint the statistically significant hotspots. We identified 31 hotspot areas for Hr-TB, further detailed information about them can be found in Supplementary Table S1. A significant portion of these hotspots was concentrated in the Minhang and Songjiang districts, as depicted in Figure 1D. When analyzing migrant and resident Hr-TB cases specifically, spatial variations were found in the migrant Hr-TB notification rate (Figure 1B) and resident Hr-TB notification rate (Figure 1C). For migrant Hr-TB cases, hotspot areas were similarly identified predominantly in Minhang and Songjiang districts (Figure 1E), areas characterized by a substantial proportion of internal migrants among the total population (Supplementary Figure S4). In contrast, for resident Hr-TB cases, hotspots were distributed in urban districts like Jingan and Hongkou, which have a dense resident population, and suburban districts like Pudong and Minhang, known for their lower resident percentages (Figure 1F).

### 3.3 Factors associated and high-risk areas with Hr-TB

Following the previous methodology, we applied stepwise regression analysis for initial variable selection in our spatial models. According to the results, we selected the percentage of older adult people (/10%), the percentage of internal migrant people (/10%), the percentage of female people (/10%), the number of healthcare institutions per 100 population, the number of health technicians per 100 population into spatial models of Hr-TB (Supplementary Table S2). In the GWR model, the AIC was estimated to be 1085.529, with an R^2^ value of 0.2215. The GWR model fit better in the southeast region in comparison to the northwest region (Supplementary Figure S5). Additional results of this model are presented in Supplementary Table S3.

**Figure 2:**
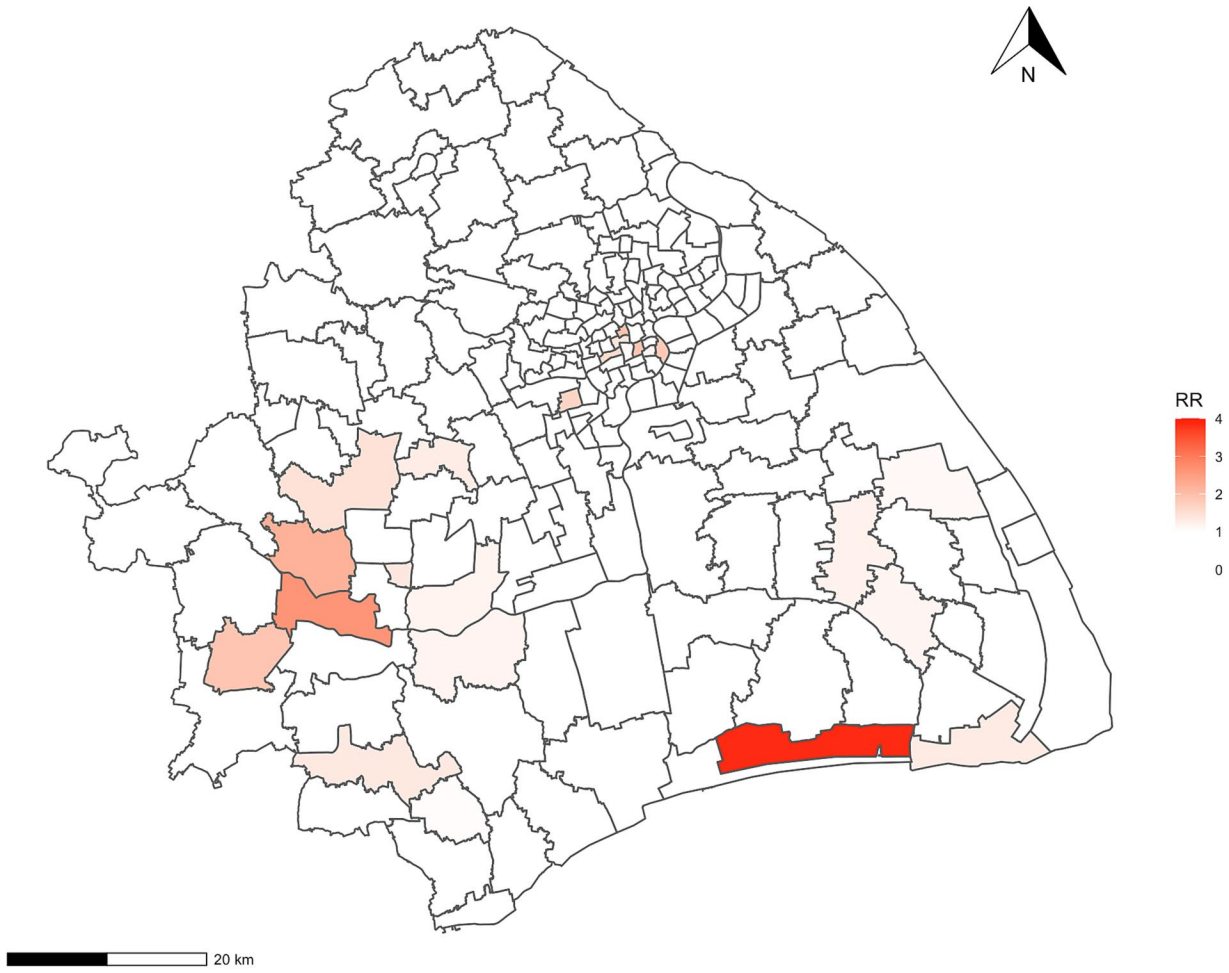
The relative-risk choropleth map for Hr-TB in Shanghai. Hr-TB: Isoniazid-resistant, rifampicin-susceptible tuberculosis; RR, relative risk.

Regarding Bayesian hierarchical models, the model that integrated spatial structured and unstructured effects was the best-fitted model for Hr-TB, as evidenced by the DIC value of 862.27 (Supplementary Table S4). The value of CPO was −2869.424 in the model. Furthermore, our analysis revealed a significant positive correlation between Hr-TB and the percentage of internal migrants (RR = 1.35, 95% Crl:1.15–1.35). Additionally, a significant positive association was observed between the percentage of older adult people and Hr-TB (RR = 3.93, 95% Crl:1.93–8.03). The number of healthcare institutions per 100 population also influenced Hr-TB rates (RR = 1.17, 95% Crl:1.02–1.34) (Table 4). Moreover, in terms of the sensitivity analysis, we found the RR results of the model remain stable even when changing the prior information of spatial effects within the model (Supplementary Tables S5, S6). Based on the Bayesian model, we unmasked that high relative risk areas were predominantly situated in the Songjiang, Fengxian, Pudong, Jingan, and Huangpu districts (Figure 2). For the urban classification, Jingan and Huangpu districts are located in the urban center of Shanghai with a high proportion of the older adult population, while Songjiang, Fengxian, and Pudong districts, mainly located in the southern part of the city, are part of Shanghai’s suburban areas. Conversely, districts with lower relative risk, such as Qingpu, Jiading, and Baoshan, are also suburban but mainly situated in the northern part of Shanghai. The county with the highest RR, named Haiwan (RR = 3.84, 95% Crl:1.05–10.06), is situated in Fengxian district, a travel region.

**Table 4:** Bayesian hierarchical model analysis to the coefficient of covariate and relative risk on Hr-TB.

	Hr TB	
	β (CrI)	RR (CrI)
(Intercept)	−4.148 (−10.086, 1.838)	0.02 (0, 6.28)
Percentage of elder people (per 10% increase)	1.37 (0.658, 2.083)	3.93 (1.93, 8.03)
Percentage of Migrants (per 10% increase)	0.301 (0.142, 0.459)	1.35 (1.15, 1.35)
Percentage of Female (per 10% increase)	0.235 (−0.969,1.427)	1.26 (0.38, 5.17)
Number of Healthcare Institutions units per 100 persons (per 1 increase)	0.157 (0.025,0.29)	1.17 (1.02, 1.34)
Number of Health Technicians per 100 persons (per 1 increase)	−0.004 (−0.008, 0.001)	1 (0.99, 1)

β, coefficient of covariate; RR, relative risk; CrI, credible interval.

## 4 Discussion

Our study analyzed the demographic characteristics and spatial distribution of Hr-TB in Shanghai. The spatial analysis revealed significant heterogeneity in the notification rate of Hr-TB at the county level in Shanghai, which was associated with the percentage of internal migrants and older adult individuals in the county. These findings underscore the importance of implementing targeted control measures in these areas with high proportions of migrants and older adult people. Moreover, we observed less favorable outcomes in Hr-TB cases when compared with DS-TB, as well as a high percentage of passive case-finding, indicating potential limitations in the management of Hr-TB patients.

This study unveiled a non-random spatial distribution of the Hr-TB notification rate. Notably, the Hr-TB hotspot areas were mainly located in the districts of Songjiang and Minhang in Shanghai. These areas are marked by a substantial internal migrant population presence. Particularly, Songjiang has a population of 1.76 million, of which internal migrants account for 61.4%, and Minhang has a population of 2.53 million, with 50.2% internal migrants (30). Interestingly, previous studies in Songjiang have highlighted the importance of recent local transmission and the impact of internal migrants on the TB burden (31, 32). And previous research has unmasked the association between high cluster areas and local transmission (33). Based on these findings, we hypothesize that a combination of factors could influence the high Hr-TB notification rate in Songjiang and Minhang. These might include the substantial internal migrant population and the direct transmission of drug-resistant *Mycobacterium tuberculosis* strains. However, a comprehensive molecular analysis is essential to substantiate the assumption.

Regarding the models, our GWR analysis may fit relatively poorly (R^2^ was 0.2215). Due to the issues for statistical inference in GWR analysis, some researchers suggested regarding Bayesian hierarchical models as an alternative to GWR (34). As a result of the modeling, we unmasked a significant positive between the percentage of internal migrants and Hr-TB. Our findings align with a previous study (15) that found the percentage of internal migrants was positively related to TB. Several factors may contribute to this observation. Firstly, the occupational practices of the migrant population often occur in a congregated setting, which may promote local transmission of TB in the population (35) and consequently increase the number of Hr-TB cases. In addition, the migrants tend to have inadequate patient adherence (36), which may lead to a higher drug-resistance rate, further promoting the emergence of Hr-TB cases. Given that Shanghai is a metropolitan area with a significant internal migrant population, special attention should be directed toward areas with a high percentage of this group in Hr-TB prevention and control efforts.

Besides, the Bayesian hierarchical model has unveiled a significant positive association between Hr-TB and the proportion of older adult individuals in Shanghai. Previous studies have reported similar results (37). This association may be due to prevalent behavioral habits among older adult individuals. For instance, older individuals often participate in congregated activities with their age group. Such congregations (e.g., game and chess rooms), especially in poorly ventilated spaces, can facilitate the transmission of *Mycobacterium tuberculosis* among attendees (38). These situations imply the importance of caring for the areas with a high proportion of older adult people.

We also noticed that the resistance rate for Hr-TB among previously treated cases in Shanghai was 15.98%, which exceeded the global rate of 11.4% (4). Additionally, our results unveiled that Hr-TB outcomes were worse compared to those of DS-TB. This observation was similar to the results of studies in other countries and regions (39–41). These findings may be due to potentially limited management strategies for TB patients (42), particularly concerning the management of previously treated cases and treatment strategies for Hr-TB. It is important to diagnose Hr-TB among previously treated cases and implement appropriate treatment strategies for them.

In our study, Hr-TB patients were mainly detected by passive pathways (95.5%, 724/758). The passive notification strategy could increase the proportion of diagnosis delay (43). Researchers found that there were a significant number of delayed diagnoses of Hr-TB within the population (10, 11); these Hr-TB patients may promote drug-resistant TB strain transmission and further progress of MDR-TB (5, 7, 8). Previous research suggested that targeting tuberculosis hotspots in disease control strategies can enhance local control efforts (44). Thus, we suggested that the key point of active screening management of Hr-TB could focus on those hotspots or areas with high-risk populations.

Our study has several limitations. Firstly, due to limited data accessibility and the retrospective nature of the analysis, certain potential individual and ecological factors associated with Hr-TB were not considered, such as smoking, drinking, and environmental conditions. Further study should analyze Hr-TB with more potential risk factors. Secondly, our study might not include all migrant TB patients in Shanghai, because some might return home for treatment. It may lead to underrepresentation in our study. However, considering the advanced medical resources in Shanghai, which could attract migrant TB patients for treatment, the impact of this limitation might not be significant. Additionally, our inference of potential Hr-TB transmission in Shanghai was based on demographic and spatial epidemiologic analysis, which lacks direct molecular evidence to support it. Future research should integrate molecular analysis to validate Hr-TB transmission. Furthermore, our findings are only applicable to Shanghai due to the lack of data from other areas for validation.

## 5 Conclusion

In conclusion, our study identified several hotspot areas for Hr-TB in Shanghai, which are mainly located in dense migrant areas. We also observed that the proportion of migrants and the proportion of older adult people were both significantly positively associated with Hr-TB, implying that areas with high ratios of these populations should be concerned. We suggested there might be a potential transmission of Hr-TB in the population, further studies with molecular analysis should be done to verify the inference. Considering Hr-TB has a potential negative impact on TB control, strategies of early detection should be implemented to prevent it from further developing worse.

## Data availability statement

The data analyzed in this study is subject to the following licenses/restrictions: the original contributions presented in the study are included in the article/Supplementary material, further inquiries can be directed to the corresponding authors. Requests to access these datasets should be directed to CY: yangchg9@mail.sysu.edu.cn.

## Author contributions

HZ: Conceptualization, Data curation, Formal analysis, Investigation, Methodology, Visualization, Writing – original draft. RS: Conceptualization, Formal analysis, Writing – original draft. ZW: Data curation, Writing – review & editing. YTL: Methodology, Writing – review & editing. MC: Investigation, Writing – review & editing. JH: Data curation, Writing – review & editing. YXL: Methodology, Validation, Writing – review & editing. FZ: Validation, Writing – review & editing. YYZ: Writing – review & editing. ML: Investigation, Writing – review & editing. HJ: Writing – review & editing. YQZ: Writing – review & editing. JX: Writing – review & editing. YX: Writing – review & editing. JY: Writing – review & editing. YZ: Writing – review & editing. XS: Data curation, Writing – review & editing. CY: Validation, Writing – review & editing.
