# Supplementary material for: Spatial pattern of isoniazid-resistant tuberculosis and its associated factors among a population with migrants in China: a retrospective population-based study
Source: Front Public Health. 2024 Mar 6;12:1372146. doi: 10.3389/fpubh.2024.1372146 (PMC10951094; doi:10.3389/fpubh.2024.1372146)
Supplement: Supplementary file 1 [file Data_Sheet_1.docx]

Supplementary Material

Spatial pattern analysis of Isoniazid-resistant Tuberculosis and its associated factors among an urban population with migrants in China

**Hongyin Zhang^1†^, Ruoyao Sun^1†^, Zheyuan Wu^2,3†^, Yueting Liu^1^，Meiru Chen^1^，Jinrong Huang^1^, Yixiao Lv^1^, Fei Zhao^4,5,6^, Yangyi Zhang^2,3,7^, Minjuan Li^1^, Hongbing Jiang^1^, Yiqiang Zhan^1^, Jimin Xu^1^, Yanzi Xu^8^, Jianhui Yuan^8^, Yang Zhao^1^, Xin Shen^2,3*‡^, Chongguang Yang^1,8,9*‡^**

*** Correspondence:**Chongguang Yang
yangchg9@mail.sysu.edu.cn

**List for supplementary material**

**Supplementary Table S1.** Information of Hr-TB hotspots in Shanghai.

**Supplementary Table S2.** Stepwise Regression Model Analysis of Hr-TB in Shanghai.

**Supplementary Table S3.** The estimated coefficient of the GWR model for Hr-TB.

**Supplementary Table S4.** Comparision of Goodness of Fit of Bayesian modles.

**Supplementary Table S5.** Sensitivity analysis of Bayesian hierarchical model by changing the prior information of spatial structured effect.

**Supplementary Table S6.** Sensitivity analysis of Bayesian hierarchical model by changing the prior information of spatial unstructured effect.

**Supplementary Figure S1.** Inclusion and exclusion of TB cases for the analysis.

**Supplementary Figure S2.** The demographic distribution of Hr-TB among new and previously treated TB cases.

**Supplementary Figure S3.** Drug resistance rate of Hr TB patients.

**Supplementary Figure S4.** Distribution map of migrant population proportion in Shanghai.

**Supplementary Figure S5.** Spatial pattern of R^2^ values in the GWR model.

**Supplementary Table S1. Information of Hr-TB hotspots in Shanghai.**

| **County** | **Town name** | **Z-score** | ***P***-value | **Gi_Bin**a | **Number of Hr-TB cases** | **Number of DS-TB cases** | **Total population** | **Migrant population** | **Resident population** |
| --- | --- | --- | --- | --- | --- | --- | --- | --- | --- |
| Fengxian | Situan town | 1.781 | 0.075 | 1 | 1 | 27 | 65389 | 23091 | 42298 |
| Xuhui | Lingyunlu | 1.805 | 0.071 | 1 | 8 | 48 | 108582 | 19938 | 88644 |
| Xuhui | Huajing town | 2.167 | 0.030 | 2 | 4 | 30 | 67415 | 28821 | 38594 |
| Xuhui | Kangjianxincun | 1.914 | 0.056 | 1 | 4 | 47 | 100444 | 25468 | 74976 |
| Xuhui | Hongmeilu | 2.202 | 0.028 | 2 | 3 | 18 | 34877 | 17377 | 17500 |
| Songjiang | Zhongshan | 3.615 | 0.000 | 3 | 5 | 99 | 98888 | 45029 | 53859 |
| Songjiang | Jiuting town | 1.701 | 0.089 | 1 | 14 | 186 | 253110 | 189398 | 63712 |
| Songjiang | Sheshan town | 2.170 | 0.030 | 2 | 6 | 73 | 75507 | 42708 | 32799 |
| Songjiang | Yexie town | 2.208 | 0.027 | 2 | 6 | 69 | 80104 | 39123 | 40981 |
| Songjiang | Xiaokunshan town | 3.096 | 0.002 | 3 | 6 | 63 | 51606 | 29960 | 21646 |
| Songjiang | Yueyang | 2.729 | 0.006 | 3 | 14 | 91 | 112671 | 24707 | 87964 |
| Songjiang | Xinqiao town | 2.263 | 0.024 | 2 | 13 | 130 | 155856 | 110254 | 45602 |
| Songjiang | Fangsong | 3.290 | 0.001 | 3 | 11 | 97 | 161438 | 52792 | 108646 |
| Songjiang | Yongfeng | 5.062 | 0.000 | 3 | 5 | 78 | 93330 | 40623 | 52707 |
| Songjiang | Maogang town | 3.062 | 0.002 | 3 | 1 | 36 | 41626 | 14312 | 27314 |
| Songjiang | Sijing town | 1.757 | 0.079 | 1 | 9 | 119 | 94279 | 68085 | 26194 |
| Songjiang | Dongjing town | 2.404 | 0.016 | 2 | 0 | 35 | 57861 | 42548 | 15313 |
| Songjiang | Shihudang town | 2.468 | 0.014 | 2 | 5 | 50 | 44011 | 24404 | 19607 |
| Songjiang | Chedong town | 3.147 | 0.002 | 3 | 23 | 233 | 167687 | 139057 | 28630 |
| Minhang | Gumei | 2.162 | 0.031 | 2 | 10 | 70 | 149141 | 47813 | 101328 |
| Minhang | Wujing town | 3.125 | 0.002 | 3 | 9 | 117 | 121164 | 62561 | 58603 |
| Minhang | Meilong town | 3.318 | 0.001 | 3 | 23 | 196 | 344434 | 186252 | 158182 |
| Minhang | Jiangchuanlu | 2.525 | 0.012 | 2 | 14 | 125 | 185991 | 38660 | 147331 |
| Minhang | Xinzhuang town | 2.371 | 0.018 | 2 | 7 | 162 | 277934 | 101058 | 176876 |
| Minhang | Zhuanqiao town | 2.212 | 0.027 | 2 | 21 | 186 | 189604 | 110011 | 79593 |
| Minhang | Maqiao town | 3.905 | 0.000 | 3 | 6 | 102 | 103989 | 72086 | 31903 |
| Qingpu | Liantang town | 2.103 | 0.035 | 2 | 1 | 24 | 68485 | 23802 | 44683 |
| Huangpu | Bansongyuanlu | 1.844 | 0.065 | 1 | 4 | 31 | 89776 | 17478 | 72298 |
| Huangpu | Waitan | 1.756 | 0.079 | 1 | 3 | 26 | 64896 | 22907 | 41989 |
| Huangpu | Laoximen | 2.532 | 0.011 | 2 | 5 | 36 | 72898 | 21816 | 51082 |
| Huangpu | Yuyuan | 2.801 | 0.005 | 3 | 2 | 20 | 61042 | 24009 | 37033 |

Abbreviations: Hr-TB: isoniazid-resistant, rifampicin-susceptible tuberculosis; DS-TB: drug-susceptible tuberculosis.

^a^Gi-bin corresponds to high-value clusters with confidence levels of 90%, 95%, and 99%, labeled as 1, 2, and 3, respectively.

**Supplementary Table S2. Stepwise Regression Model Analysis of Hr-TB in Shanghai.**

| Variates | Estimate | Standardized ErrorError | t value | *P* value | Tolerance Value |
| --- | --- | --- | --- | --- | --- |
| Constant | -32.04997 | 8.86889 | -3.614 | <0.001 |  |
| Percentage of elder people (per 10% increase) | -4.80708 | 1.25111 | -3.842 | <0.001 | 3.43392 |
| Percentage of internal Migrants (per 10% increase) | 0.69174 | 0.25711 | 2.690 | 0.008 | 2.75266 |
| Percentage of Female (per 10% increase) | 7.92515 | 1.85572 | 4.271 | <0.001 | 2.81580 |
| Number of Healthcare Institutions units per 100 population (per 1 increase) | 0.59084 | 0.23168 | 2.550 | 0.012 | 3.75598 |
| Number of Health Technicians per 100 population (per 1 increase) | -0.01789 | 0.00787 | -2.275 | 0.024 | 3.76627 |

Abbreviations:Hr-TB: isoniazid-resistant, rifampicin-susceptible tuberculosis.

**Supplementary Table S3. The estimated coefficient of the GWR model for Hr-TB.**

| Variable | Minimum | 1st Quartile | Median | 3rd Quartile | Maximum |
| --- | --- | --- | --- | --- | --- |
| Constant | -32.402761 | -32.214281 | -32.162935 | -32.129771 | -31.9401 |
| Percentage of elder people (per 10% increase) | -4.864605 | -4.837675 | -4.828975 | -4.823186 | -4.809262 |
| Percentage of internal Migrants (per 10% increase) | 0.687757 | 0.690819 | 0.692249 | 0.694653 | 0.703712 |
| Percentage of Female (per 10% increase) | 7.913985 | 7.945788 | 7.952802 | 7.964041 | 8.005409 |
| Number of Healthcare Institutions units per 100 population (per 1 increase) | 0.588868 | 0.592462 | 0.593301 | 0.593824 | 0.594945 |
| Number of Health Technicians per 100 population (per 1 increase) | -0.018067 | -0.017942 | -0.017915 | -0.017898 | -0.017807 |

**Supplementary Table S4. Comparision of Goodness of Fit of Bayesian modles.**

| **Model** | **DIC** | **WAIC** |
| --- | --- | --- |
| Non-spatial effect model | 1184.52 | 1208.88 |
| Spatial structured effect model | 867.77 | 883.61 |
| Spatial unstructured effect model | 862.63 | 871.49 |
| Spatial structured and unstructured effect model | 862.27 | 871.05 |

Abbreviation: DIC, Deviance Information Criterion; WAIC，Watanabe-Akaike information criterion.

|  | loggamma(1,0.01) | loggamma(1,0.001) | loggamma(1,0.0001) |
| --- | --- | --- | --- |
|  | RR (CrI) | RR (CrI) | RR (CrI) |
| (Intercept) | 0.01（0,3.14） | 0.01, (0,3.13) | 0.01, (0,3.11) |
| Percentage of elder people (per 10% increase) | 3.93 (1.93,8.03） | 3.93, (1.93,8.01) | 3.93, (1.93,8.02) |
| Percentage of Migrants (per 10% increase) | 1.35（1.15,1.58） | 1.35, (1.15,1.58) | 1.35, (1.15,1.58) |
| Percentage of Female (per 10% increase) | 1.26 (0.38,4.17） | 1.26, (0.38,4.16) | 1.26, (0.38,4.16) |
| Number of Healthcare Institutions units per 100 persons (per 1 increase) | 1.17（1.02,1.34） | 1.17, (1.02,1.34) | 1.17, (1.02,1.34) |
| Number of Health Technicians per 100 persons (per 1 increase) | 1（0.99,1） | 1, (0.99,1) | 1, (0.99,1) |

**Supplementary Table S5. Sensitivity analysis of Bayesian hierarchical model by changing the prior information of spatial structured effect.**

Abbreviations: β, coefficient of covariate; RR, relative risk; CrI, credible interval.

**Supplementary Table S6. Sensitivity analysis of Bayesian hierarchical model by changing the prior information of spatial unstructured effect.**

|  | loggamma(1,0.01) | loggamma(1,0.001) | loggamma(1,0.0001) |
| --- | --- | --- | --- |
|  | RR (CrI) | RR (CrI) | RR (CrI) |
| (Intercept) | 0.01, (0,3.1) | 0.01, (0,3.11) | 0.01, (0,3.14) |
| Percentage of elder people (per 10% increase) | 3.93, (1.93,8.02) | 3.93, (1.93,8.02) | 3.93, (1.93,8.03) |
| Percentage of Migrants (per 10% increase) | 1.35, (1.15,1.58) | 1.35, (1.15,1.58) | 1.35, (1.15,1.58) |
| Percentage of Female (per 10% increase) | 1.26, (0.38,4.16) | 1.26, (0.38,4.16) | 1.26, (0.38,4.17) |
| Number of Healthcare Institutions units per 100 persons (per 1 increase) | 1.17, (1.02,1.34) | 1.17, (1.02,1.34) | 1.17, (1.02,1.34) |
| Number of Health Technicians per 100 persons (per 1 increase) | 1, (0.99,1) | 1, (0.99,1) | 1, (0.99,1) |

Abbreviations: β, coefficient of covariate; RR, relative risk; CrI, credible interval.


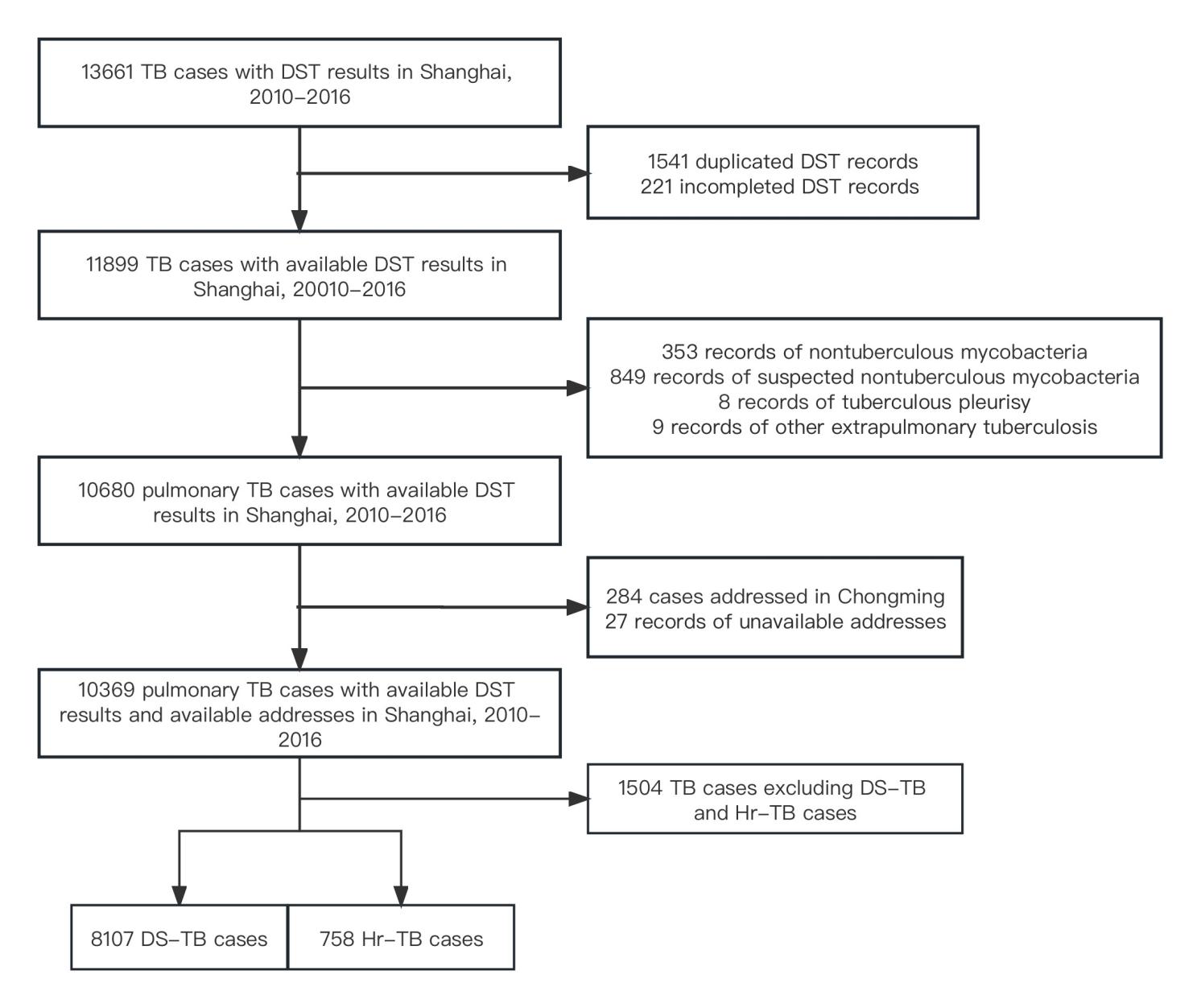


**Supplementary Figure S1. Inclusion and exclusion of TB cases for the analysis.**

Abbreviations: TB, tuberculosis; DST: Drug Susceptibility Tests; DS-TB: drug-susceptible tuberculosis; Hr-TB: isoniazid-resistant, rifampicin-susceptible tuberculosis.


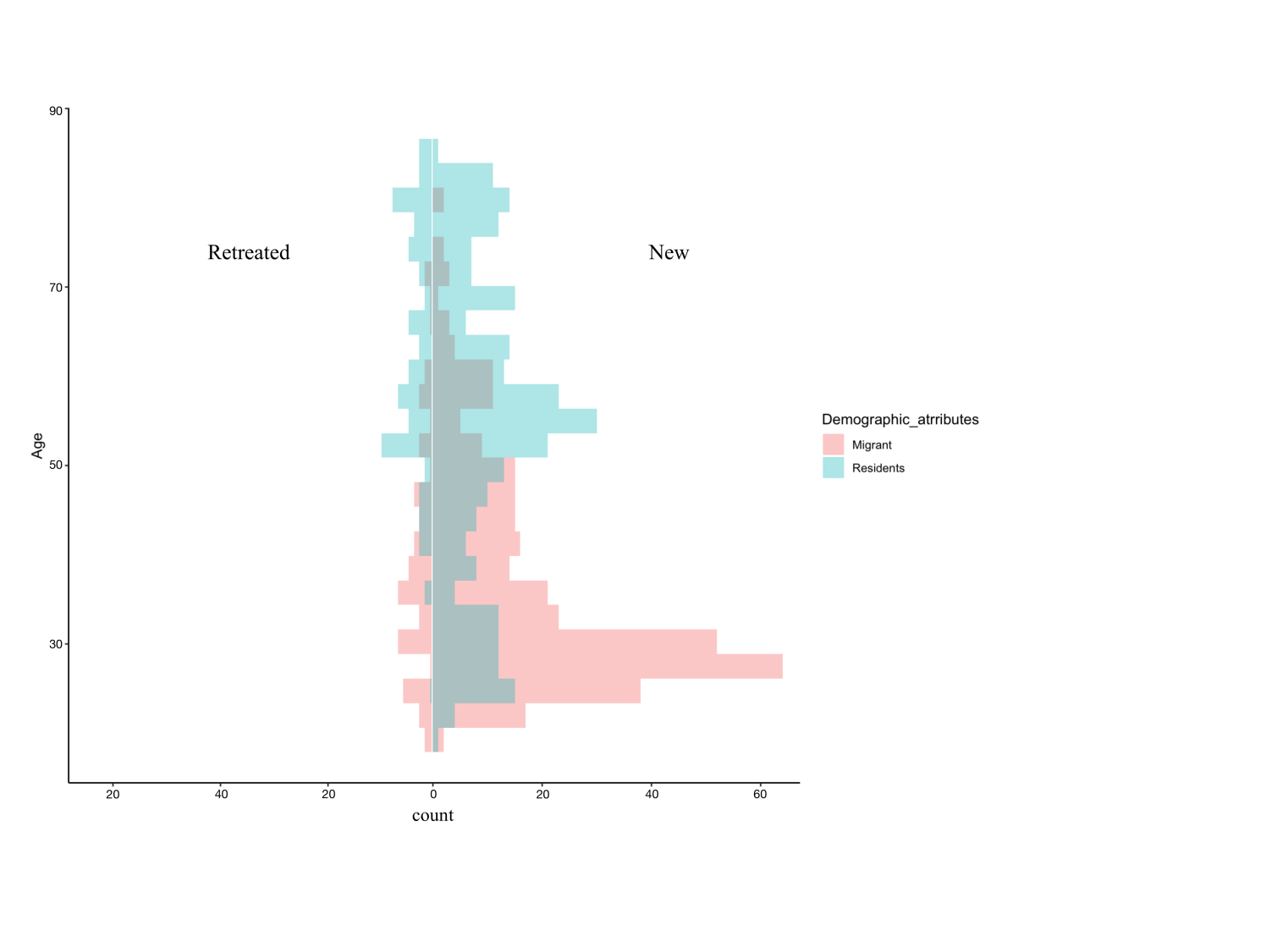


**Supplementary Figure S2. The demographic distribution of Hr-TB among new and previously treated TB cases.**

Abbreviations: Hr-TB: isoniazid-resistant, rifampicin-susceptible tuberculosis.


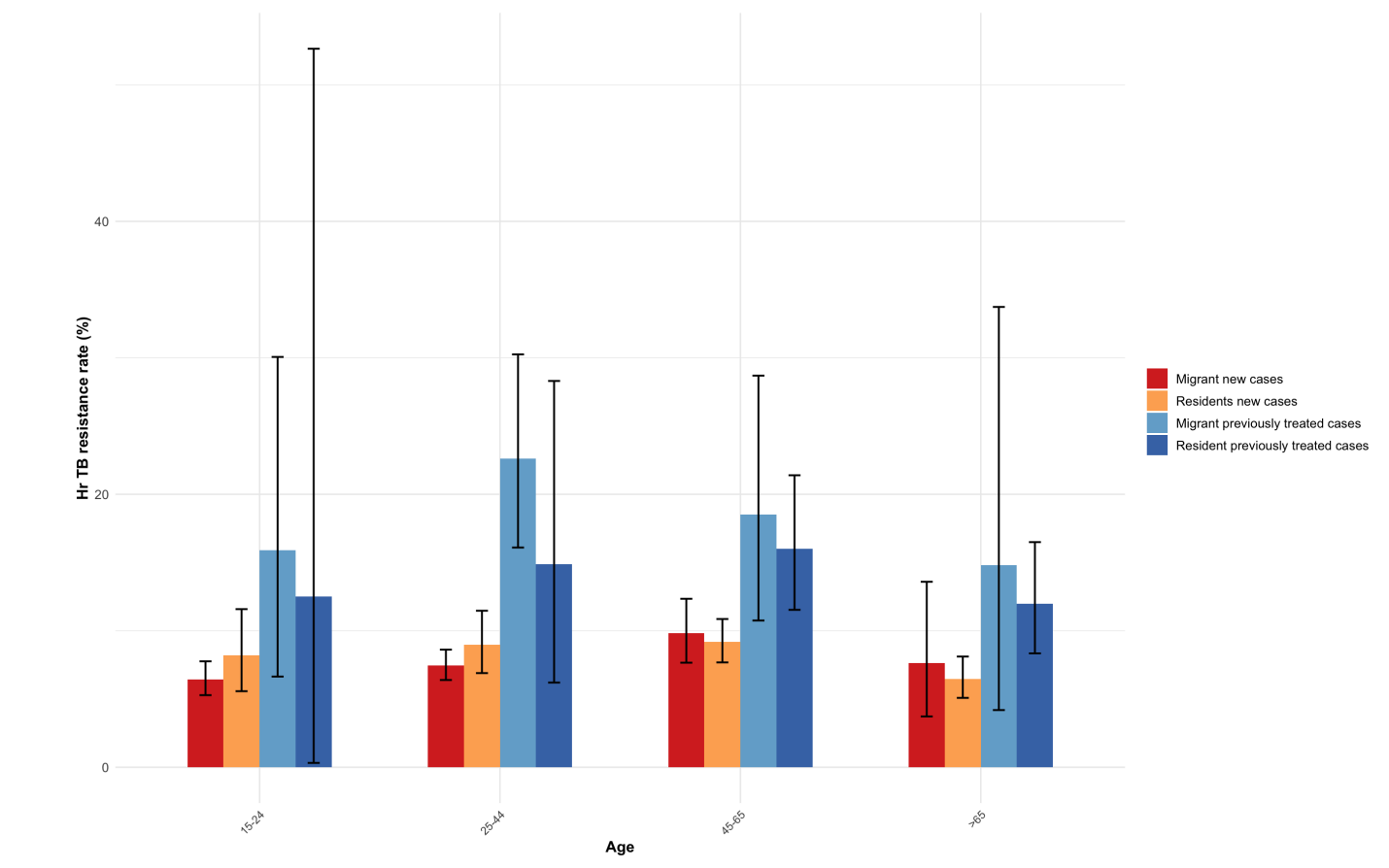


**Supplementary Figure S3. Drug resistance rate of Hr-TB patients.**

Abbreviations: Hr-TB: isoniazid-resistant, rifampicin-susceptible tuberculosis.


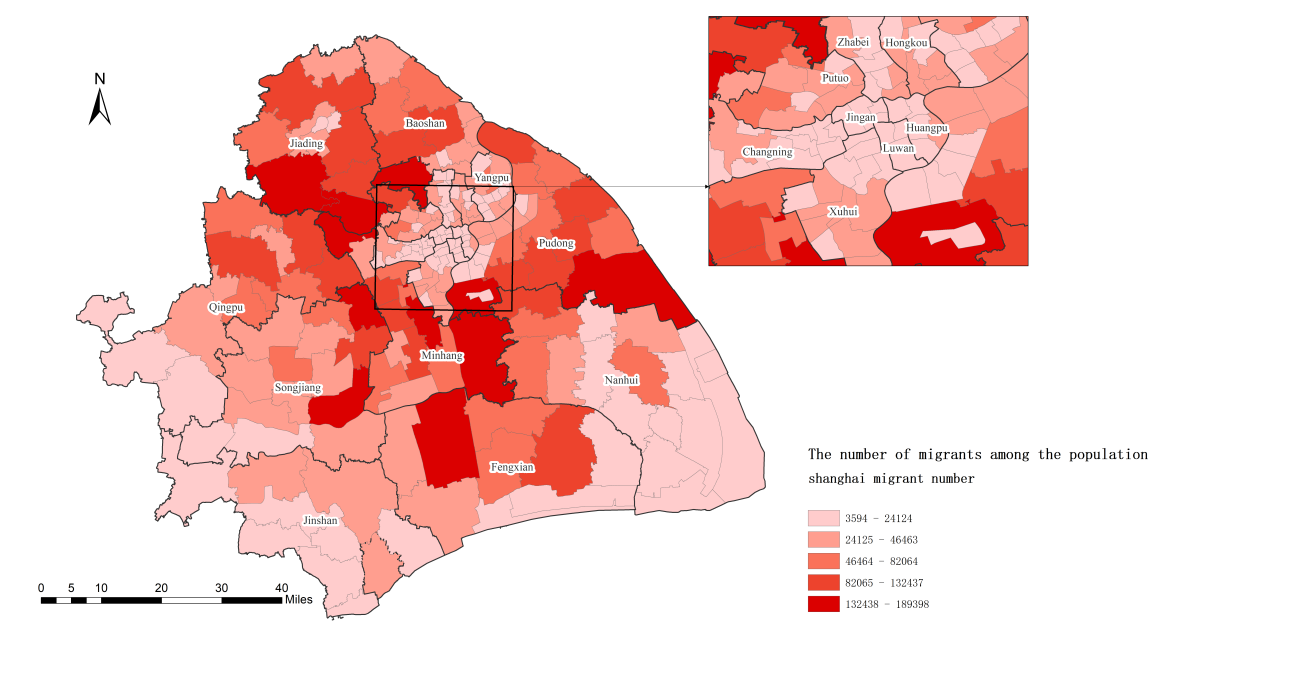


**Supplementary Figure S4. Distribution map of migrant population proportion in Shanghai.**

**
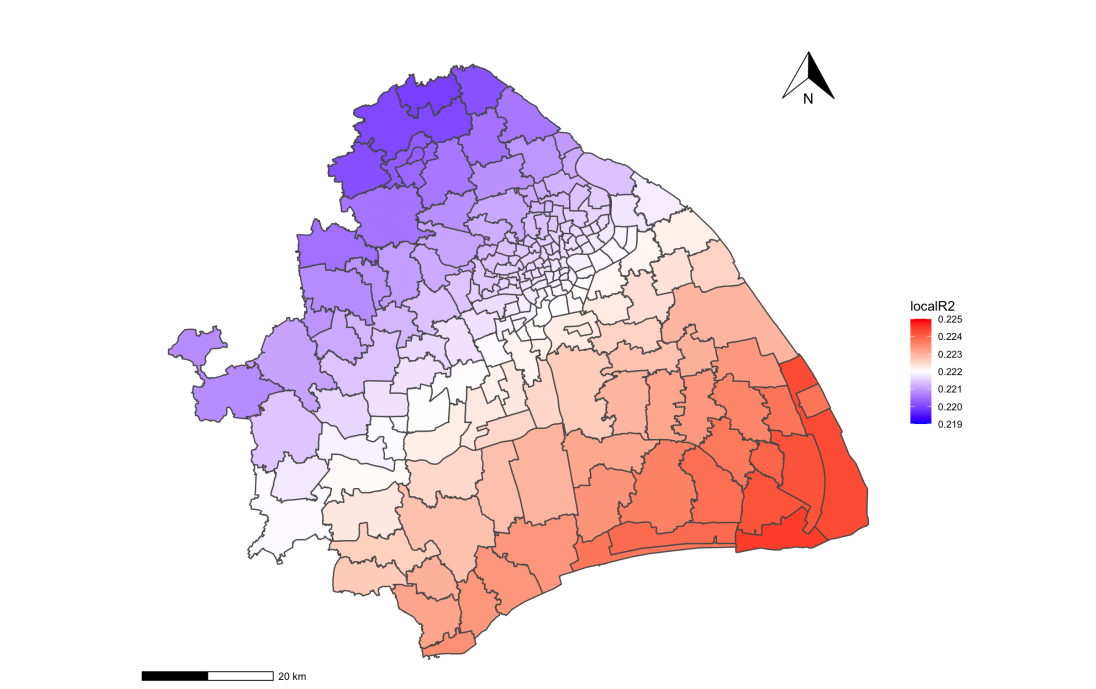
**

**Supplementary Figure S5. Spatial pattern of R^2^ values in the GWR model.**

The map shows R^2^ values changing from low (blue) to high (red).
